# Entropium Proved to Depend on Muscular Action

**Published:** 1852-09

**Authors:** Haynes Walton

**Affiliations:** Surgeon to the Central London Ophthalmic Hospital, and to St. Mary’s Hospital, Paddington


*Entropium Proved to Depend on Muscular Action.* By HAYNES WALTON, ESQ., F. R. C. S., Surgeon to the Central London Ophthalmic Hospital, and to St. Mary's Hospital, Paddington.—I have founded the treatment on what appears to me to be the pathological interpretation of the affection, and of which the indications are, to overcome the means of inversion by dissecting away the thick marginal portion of the orbicularis, supposing that part of the muscle to be entirely, or nearly all that is at fault, and also to remove as much of the skin of the lid as may be required by its loss to produce such tension as shall overcome and restore to a natural state, whatever unnatural position the other tissues or component parts of the lid may have acquired, from the irregular position into which they have been thrown by the muscle, and which has been made more or less permanent by such changes as inflammation would produce.

Now, as to the manner of operating: let us suppose that the right eye is to be done. An assistant stands behind the patient, and having made the lid tense by drawing it outwards and raising the brow, two incisions are made through the skin and muscle, one along the edge of the tarsus close to the cilia, and the second about the quarter of an inch above, and meeting the other at the extremities. The flap thus isolated should be dissected vertically from the one side to the other, and not taken away by horizontal strokes of the knife, or else the muscular portion will not be effectually removed. The wound should be very carefully sponged during the operation. Any arterial jet must be checked by temporary pressure with the finger. I have never found a ligature to be necessary. The exposed surface must be inspected, and, if any muscular fibres have escaped, the forceps and knife must be re-applied. The assistant should not desist until the knife has been laid aside, for the proper retraction of the skin is essential to steady and effectual dissection. Three or four sutures should be used. The cilia might appear to be in danger of being dissected off, but in reality they are not. A part only of the dissection is over them, and by the loose cellular connexion, the muscle is readily raised from the dense fibro-cellular tissue in which they lie.

The previous observations almost demand a cursory view of the usual methods of operating, and their results; indeed, I think this paper very imperfect without it; yet I fear that its introduction would make my communication too long. I must be satisfied with making the assertion, that there is not any known method of operating for entropium, that can be depended on, except that of cutting off the greater portion of the lid, and which is as bad in principle,—nay, even worse, if the physiological relations of the lid be taken into account,—as to amputate a thigh to cure a popliteal aneurism.

Heretofore, when an operation for entropium has been undertaken with the design of removing any part of the orbicularis palpebrarum muscle, it has been executed on very different principles, and in a very different manner to that I now advocate, and with very different results. The marginal part of the muscle, the musculus ciliaris, has been untouched, and only a bit of the centre of that on the lid has been snipped out. The only exception I know of is in the practice of Mr. Key. I am astonished that the notice of the operation he performed, and which is in the *Lancet* for 1825, page 235, should have escaped the attention of our writers on ophthalmic surgery. It is very lately that I have been acquainted with it, long after I had matured my own views. I cannot say whether Mr. Key continued to execute it, and what were his ideas on the subject in later years. The report runs thus: Mr. Key considered the inverted state of the tarsus to arise from the action of the orbicularis palpebrarum muscle. With that view he determined on laying bare the substance of the lower tarsus, and dissecting off the fibres of the orbicularis. The operation was performed by first turning out the lid, and then making an incision through the skin along the whole length of the lower eyelid, at a few lines distance and below the ciliary ridge; the integuments were carefully elevated by means of dissecting forceps, and the fibres of the orbicularis thus exposed were as carefully removed; there was considerable bleeding from the parts; the portion of skin which had been raised was laid down, and the wound dressed by means of adhesive straps, with a compress applied on the skin. The result is said to be successful. Mr. Tyrrell and Mr. Travers had operated previously, the first by cauterisation, the second by removing a portion of skin without success.

M. Desmarres has not overlooked the influence of the orbicularis in producing the inversion, and he gives a case that he thinks solely due to it; but he does not recognize its general operation, and he significantly indicates how little he appreciates the principle of Mr. Key's execution, and the triumphant issue of the case, by asking, in a commentary on the proceeding, "Is it to the incision of the skin, or the excision of the muscle, that the success is owing?" Spasmodic action of the muscle is spoken of by foreign authors, but with much difference of opinion concerning its frequency in producing entropium. The usual Continental mode of operating on the orbicularis is subcutaneous incision, which seems to have been unsuccessful; that of M. Janson de Lyon is a terrible affair, the cutting out of several vertical portions of skin, including some fibres of the orbicularis. Dr. Mackenzie, in his only notice of operating on the muscle, informs us, that it may be proper, after removing the cutaneous fold, to snip off a few fibres, so as to form a firmer cicatrix, actually fixed to the cartilage; and Mr. Lawrence, a much later author, echoes the same thing, for he writes, "In an incipient case, it may be sufficient to excise a portion of skin; this remedy will, at least, answer the purpose foı a time. To make the operation more effectual, a portion of the orbicularis should be removed also, that a firmer cicatrix may be produced; or the acid may be employed, using it more freely, so that its action may extend deeper, and a solid scar be the result."

A narration of cases, with the various symptoms and terminations of entropium, must be excluded from this paper, which is intended to embrace only general principles.

As trichiasis commonly exists with entropium, and the operation I have advocated bears on the united affections, I shall say a few words relative to it.

With inversion of the upper lid, there is not unfrequently decided trichiasis, and, whether the two are due to the same exciting cause, or the trichiasis have been the original affection, or merely an effect of the inversion, it matters nothing as far as the treatment is concerned; yet it may not be amiss to remark, that, with severe entropium, the edge of the lid can rarely for any length of time escape from being affected by the general inflammation, and that the cilia will the more readily retain any unnatural position in which they may be placed. And I am inclined to regard the trichiasis more frequently as an effect, because it is generally of one form, that of a separation or twisting in of the innermost of the cilia from their fellows, and that without any alteration or degeneration in the individual hairs, and because the removal of the entropium is generally sufficient to clear the globe of them. Although, practically speaking, such a degree of trichiasis matters little, it is important, before operating for the entropium, to ascertain whether there is also trichiasis, and whether the restoration of the lid to its natural position will or will not counteract the mal-direction of the cilia. Should it not be sufficient, then more skin must be taken from the lid than would otherwise have been necessary, and a slight degree of eversion of the centre of its edge produced, and which must necessarily have its limit.

When it is apparent that such moderate eversion will not suffice, the treatment must depend on the degree of the trichiasis; if it be general, the entropium and it must be attacked by one operation. The cilia must be excised at the same time, that a sufficient extent of the skin of the muscle should be removed. But when the trichiasis is partial—and, for the most part, it is—the muscle should first be dissected away, and then the irregular cilia removed. When there is doubt about the necessity of removing them, attend to the entropium alone, and observe the result, because it is not always possible before the operation for the entropium has been done, and the lid recovered from it, as well as from any inflammation and swelling that the entropium may have induced, to ascertain with exactness to what extent the trichiasis may be benefitted. If, as is commonly the case, a patient applies to be treated for entropium, with many of the cilia broken, and some just about to be restored after having been plucked out, it cannot be known what direction they may assume when growing out; and such cases should be watched.

It can be very seldom that the removal of entropium from the lower lid does not at the same time separate any irregular cilia from contact with the globe, for a single exception only has occurred to me.—*Dublin Medical Press.*

